# A "Gunpowder Plot"

**Published:** 1920-10-23

**Authors:** 


### A "Gunpowder Plot."

We can profess little admiration for medical men who, in grotesquely picturesque terms, set forth views which are contrary to generally accepted facts. Dr. J. R. Leeson, a member of the Middlesex County Council, has been indulging in extravagant and wholesale condemnation of milk as "a deadly drink," and declaring that he would "rather see a cask of gunpowder than a glass of milk in a nursery." So far as can be gathered from his statement to an interviewer, his objections to milk are based upon defects in the supply of properly produced and properly distributed milk, but yet he recommends water without the slightest hint of any consideration of the defects of water supplies. What a vision of strong, vigorous and healthy babes is conjured up by regular feeds of plain water from birth and during infancy! Has Dr. Leeson experimented, and if not, upon what does he base his assertion? It is no argument for Dr. Leeson to point to the number of deaths from tuberculosis, or to show what damage infected milk can do. Those points are news to nobody. What is needed is a persistent exhortation of the value of rich, pure milk, and not an exhortation to foolish people to ignore its life-building properties. There are many differences among medical men regarding the different values of food, or the laws of health, and expert views have changed from generation to generation, often to the perplexity of ordinary folk. But views on milk have remained constant, and all efforts are turned towards making a pure supply accessible to everybody. Lord Bledisloe, the President of the British Farmers' Association, speaking at the annual show this week declared that the public has been discouraged from drinking milk by alarmist statements of its impurities; he agrees there is scope for considerable improvement, but contends, what is undoubtedly true, that the milk consumed by the British public to-day is infinitely better and more free from disease than the milk of a generation ago.

